# Correlation Between Manufacturing Conditions, Microstructure, and Electrical–Mechanical Properties of Cu Matrix Composites

**DOI:** 10.3390/ma19071347

**Published:** 2026-03-28

**Authors:** Marko Simić, Emilija Nidžović, Svetlana Butulija, Željko Radovanović, Marija M. Vuksanović, Jovana Ružić

**Affiliations:** 1 “Vinča” Institute of Nuclear Sciences-National Institute of the Republic of Serbia, University of Belgrade, 11001 Belgrade, Serbia; marko.simic@vin.bg.ac.rs (M.S.); emilija.nidzovic@vin.bg.ac.rs (E.N.); svetlana8@vin.bg.ac.rs (S.B.); marija.vuksanovic@vin.bg.ac.rs (M.M.V.); 2 Innovation Center of the Faculty of Technology and Metallurgy, Ltd., Karnegijeva 4, 11120 Belgrade, Serbia; zradovanovic@tmf.bg.ac.rs

**Keywords:** Cu–matrix composites, powder metallurgy, mechanical alloying, mechanical properties, conductivity

## Abstract

The continuous demand for advanced composite materials with superior mechanical and electrical properties has driven the exploration of copper matrix composites for high-performance applications. The Cu–2Zr–0.6B (wt.%) powder mixtures were mechanically alloyed (MA) using two different ball-to-powder weight ratios (BPR: 10:1 and 15:1) to investigate the influence of milling conditions on the final composite material’s properties. MA powders milled with BPR 15:1 exhibited the highest values of dislocation densities, which induce higher hardness of Cu–ZrB_2_ bulk materials. The MA powders were consolidated using three different methods: conventional cold pressing followed by sintering (CPS), hot pressing (HP), and spark plasma sintering (SPS). The in situ forming of ZrB_2_ (3.5 vol.%) reinforcements during consolidation processes in Cu matrix proved to have a major impact on enhancing the hardness and structural stability, while the use of SPS and HP offered superior control over grain growth and porosity reduction compared to CPS. Main findings related to electrical and mechanical properties showed similar values for SPS (~38% IACS, ~173 HV1) and HP compacts (~39% IACS, ~155 HV1) but proved to be much higher compared to values of CPS compacts (~21% IACS, ~80 HV1).

## 1. Introduction

With a growing demand for new, enhanced composite materials that can be used in various industries such as electrical–electronics, transportation, aerospace, military, and automotive, new materials are constantly being developed using different techniques [1,2,3,4]. Copper matrix composite materials have been identified as promising materials in these industries for various use cases, such as electrical contacts and connectors in the electrical–electronics [5], heat sinks and thermal management systems in the aerospace industry [5], and field coils and stabilizers for superconducting coils in magnetic confinement thermonuclear fusion reactors [6], to name a few. The main requirement for these demanding working conditions is the balance between strength/hardness and electrical conductivity. Hence, adequate selection of the manufacturing process of copper-based composites is crucial for reaching this balance. The multiple advantages that powder metallurgy (PM) offers compared to other manufacturing methods include improved material properties and reduced waste generation [7]. For Cu–Zr–B systems, the in situ formation and uniform distribution of fine ZrB_2_ particles within the copper matrix is facilitated, which is difficult to achieve through casting techniques. Powder metallurgy allows the production of composites with refined microstructures and improved mechanical properties while maintaining relatively high electrical and thermal conductivity [7]. One of the main advantages also stems from the ability to precisely control the chemical composition and microstructure of the material, which enables the design of materials with tailored mechanical and physical properties. Furthermore, PM processing typically occurs at temperatures below the melting point of the base material, which helps prevent undesirable reactions and segregation that are often observed in casting processes [8].

For Cu–matrix composite materials, mechanical alloying (MA) as a PM technique plays an important role in improving their properties. As a result of the formation of a highly disordered microstructure during the process, mechanical properties are optimized [9]. As there are still limitations that conventional processing techniques cannot get past, the MA process proves to be an essential technique in powder metallurgy, because it enables the development of materials with distinctive properties [10]. High-energy ball milling induces intense plastic deformation in the materials, resulting in the development of a highly disordered microstructure [11]. This structural refinement can lead to improved mechanical properties, including increased hardness, ductility, and strength [12].

Although Cu–ZrB_2_ composites fabricated by powder metallurgy and spark plasma sintering (SPS) have been reported in the literature, most recent studies primarily focus on optimizing a single processing route or evaluating the final mechanical properties of the composites. Several recent studies have explored Cu–matrix composites reinforced with zirconium diboride using powder metallurgy and spark plasma sintering techniques. Sulima [13] and Li [14] fabricated Cu–ZrB_2_ composites by ball milling and SPS, showing that the addition of ZrB_2_ significantly increases hardness (from ~130 to final ~250 HV) and elastic modulus, although electrical conductivity decreases with increasing reinforcement fraction (from 30 to final 15% IACS). More recent investigations have also examined the influence of ZrB_2_ content and sintering parameters on the microstructure and tribological properties of SPS-consolidated Cu composites sintered at 850 °C and above [15]. Additionally, Li et al. [14] reported that boride reinforcements such as ZrB_2_ can effectively strengthen Cu–matrix composites while influencing their electrical transport behavior depending on the microstructural distribution of the ceramic phase. Systematic comparisons between different consolidation techniques and their influence on the processing–structure–property relationship remain limited.

The Cu–matrix composite materials reinforced with diborides (TiB_2_ and ZrB_2_) are often chosen for demanding applications because of their prized properties, such as high hardness, excellent electrical and thermal conductivity, improved fatigue strength, great thermal stability, and enhanced wear resistance [1,12,16]. The ratio of the constituents (Ti or Zr) as well as the selection of the processing technique plays a big role in the final properties of these composite materials [11]. Taking the constituents’ ratio into account, the addition of boron into the copper matrix can improve the mechanical properties of the composite material by in situ forming diborides, which act as physical barriers to dislocation motion [17,18]. On the other hand, diborides have lower conductivity than the copper matrix and act as scattering centers. Apart from the boride reinforced materials, recent studies also focus on other families of these materials, such as oxides, but borides are preferred due to overall better electrical conductivity achieved [19]. By a closer look at composite materials that are required to exhibit high strength and high electrical/thermal conductivity, there is always going to be a trade-off between the two required properties [18]. For that reason, research focused on the development of the high-performance Cu–ZrB_2_ composites, as perfect candidates to tackle this industry-wide problem, is being strongly encouraged [20].

In this study, powder metallurgy techniques were employed for manufacturing copper matrix composites reinforced with about 3.5 vol.% ZrB_2_. Three different routes (mechanical alloying followed by: (1) cold pressing and sintering, (2) hot pressing, and (3) spark plasma sintering) for Cu–ZrB_2_ composite material production were observed. Mechanical alloying was carried out for 20 h with two different ball-to-powder ratios (10:1 and 15:1). The technological parameters for mechanical alloying were selected based on commonly reported conditions in the literature for Cu-based composite powders, where similar ball diameters and ball-to-powder ratios are frequently used to ensure effective energy transfer during milling. Two ball-to-powder ratios (10:1 and 15:1) and different ball size configurations were examined in order to evaluate the influence of milling energy and collision frequency on particle refinement and microstructural evolution during high-energy mechanical alloying. The selected milling time and stirring speed were chosen to provide sufficient mechanical activation while avoiding excessive agglomeration or contamination of the powder. Furthermore, the consolidation parameters were selected to represent three of the most commonly used powder metallurgy routes, which allowed a direct comparison of their influence on densification behavior and the resulting microstructure and properties of the Cu–ZrB_2_ composites.

Taking that into account, the aim of the present study is to show the effect that mechanical alloying parameters coupled with selected consolidation techniques have on the electrical and mechanical behavior of the obtained composite materials. The influence of the microstructural parameters of mechanically alloyed powders (Cu–2Zr–0.6B (wt.%)) and their compacts (Cu–ZrB_2_) on the final materials’ properties was also examined.

## 2. Materials and Methods

Copper, zirconium, and boron powders with particle sizes of approximately 30 µm (99.5% purity) from manufacturer Pometon TIR Ltd., Bor, Serbia, 2 µm (99.5% purity) from manufacturer Herman C. Starck, Berlin, Germany, and 0.08 µm (97% purity) from manufacturer Heraeus, Hanau, Germany, respectively, were used as starting materials. The powder mixture composition Cu–2Zr–0.6B (wt.%) was set to ensure in situ formation of 3.5 vol.% ZrB_2_ during consolidation processes. This amount of the reinforcing phase was chosen as a compromise between improving the mechanical strength and preserving the high electrical and thermal conductivity of the copper matrix. It is well known that increasing the volume fraction of a ceramic phase leads to stronger reinforcement, but at the same time significantly reduces the electrical conductivity of the composite [1]. On the other hand, a very low amount of reinforcement does not provide a sufficient contribution to mechanical strengthening [2]. Therefore, a content of approximately 3.5 vol.% ZrB_2_ allows a measurable increase in hardness and strength while maintaining conductivity at a level acceptable for highly conductive copper-based composites.

The starting powders were first homogenized in the Turbula Shaker Mixer Type T2C (Artisan Technology Group, Basel, Switzerland) for 1 h. Homogenization was followed by mechanical alloying (MA) in the same Turbula mixer mill with process parameters: stainless steel balls with 6 mm diameter, ball-to-powder wt. ratio (BPR) 15:1 and 10:1, inert argon atmosphere, alloying time 20 h, and stirring speed 330 rpm.

Three different methods of the MA powders’ consolidation were employed: (1) Cold pressed at 350 MPa (5 min holding time) and then sintered in the Protherm PTF 16/38/250 tube furnace (Ankara, Turkey) at 1030 °C for 2 h in argon atmosphere. (2) Sintered in the Dr Fritsch DSP 507 (Fellbach, Germany) sintering press at 750 °C for 5 min at a pressure of 35 MPa in vacuum. The initial load applied, heating/cooling regime, and dwell time (8 MPa, 100 °C/min, 50 °C/min, 5 min, respectively) were constant for all the samples. (3) Hot pressed in Astro furnace at 950 °C for 2 h at a maximum load of 40 MPa in argon atmosphere with heating speed of 15 °C/min. Abbreviations used for each sample are listed in Table 1 for clarity and easier reference. All samples were the same cylindrical shape with different diameters. Samples (1) and (2) from Table 1 have a diameter of 20 mm and a height of 5 mm. Samples (3), (4), (5), and (6) have the same diameter of 12 mm and height of 4 mm. The number of tested samples was three across all three consolidation methods.

The specific surface area (SBET) of both Cu-2Zr-0.6B (wt.%) 10:1 (ball: powder ratio) and Cu–2Zr–0.6B (wt.%) 15:1 (ball: powder ratio) MA powders was determined from N_2_ adsorption–desorption isotherms measured at −196 °C using a Surfer analyzer (Thermo Fisher Scientific, Waltham, MA, USA). Prior to analysis, the samples were degassed at 120 °C for 24 h under vacuum. SBET values were calculated using the Brunauer–Emmett–Teller (BET) equation.

MIPAR image analysis software (version 4.5) was used to do the quantitative analysis of the SEM images of the MA powders that were previously taken. X-ray diffraction (XRD) was utilized in order to assess the samples’ phase composition and structural parameters, investigating both powders and compacts. The analysis was conducted using a RIGAKU Ultima IV (Tokyo, Japan) diffractometer with Ni-filtered Cu Kα radiation (λ = 0.1540 nm). The used generator voltage and current were 40 kV and 40 mA, respectively. Continuous scanning was done over the 20–80° 2θ range, with a step of 0.02° and a scanning speed of 10°/min. PDXL2 software (version 2.0.3.0) [21] with reference to the International Centre for Diffraction Data was used to determine the samples’ phase composition and structural parameters. The Williamson–Hall method was employed to estimate the crystallite size (*D*, A), lattice parameter (*a*, A), and lattice strain (ε, %) of the mechanically alloyed powders and samples. Dislocation density (ρ, m^−2^) was calculated using the relation ρ $=\frac{\varepsilon \times 2\times \sqrt{3}}{D\times b}$, where b is the magnitude of the Burgers vector (0.255 nm for Cu [22,23,24]. Feld emission scanning electron microscope MIRA3 TESCANXMLI, Oxford, UK (SEM) fitted with Energy Dispersive X-ray Diffraction Spectroscopic Analyzer (EDS) was employed to assess how mechanical alloying parameters affected the microstructure and morphology of the Cu–Zr–B powder mixtures and corresponding compacts.

Density measurements of all sintered samples were conducted using Archimedes’ method in water (analytical balance), while the theoretical density was calculated using the rule of mixtures. Open porosity was determined using Archimedes’ method. The dry mass of the sample (m_1_), the saturated mass in air (m_2_), and the apparent mass while immersed in water (m_3_) were measured. Open porosity was calculated according to the following relation: $P_{open\;}(\%)=\frac{m_{2}-m_{1}}{m_{2}-m_{3}}\times 100$.

The Vickers macrohardness of the hot-pressed and cold-pressed-sintered treated samples was measured using a Buehler hardness tester (Lake Bluff, IL, USA) under a 1 kg load. The nanohardness and reduced elastic modulus of the compacts were measured using a TI 950 Hysitron Triboindenter equipped with a Berkovich indenter (Minneapolis, MN, USA) under the maximum applied load of 3 mN, with 10 s dwell at maximum load, and constant loading and unloading rates of 300 µN/s. Data was collected from 12 indents for each compact, and both macro and nanoindentation tests.

**Figure 1:**
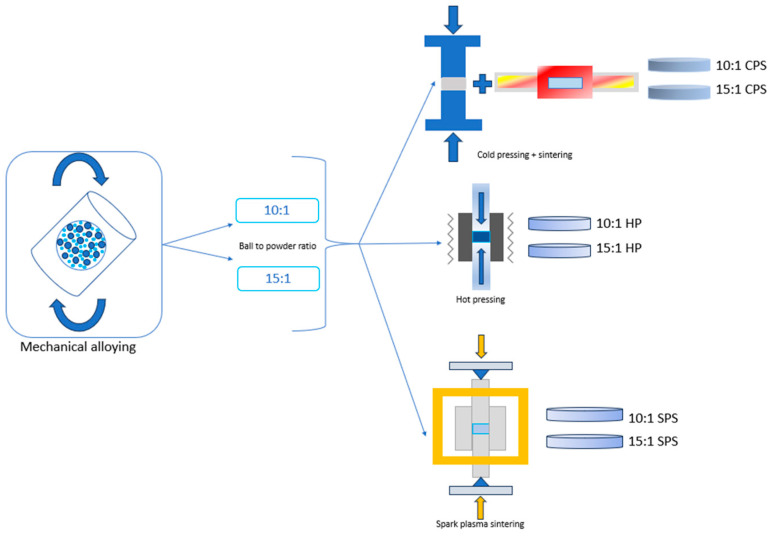
Flowchart showing the process that was followed in this paper. Mechanical alloying, different MA process parameters, and three consolidation techniques: CPS, HP, and SPS.

Electrical conductivity (σ) was tested on the device Forester Sigma Test 2069 (Reutlingen, Germany), at a frequency of 240 kHz. The conductivity values were converted to the International Annealed Copper Standard (% IACS) using the formula:$\;\%IACS=\;\sigma \times 1.7241\times 10^{-6}\;$[25]. Based on the measured values of electrical conductivity, the thermal conductivity was also calculated using the Wiedemann–Franz law, according to the relation: σ×k=T×L, where k is the thermal conductivity (W m^−1^ K^−1^), σ is the electrical conductivity (Ω^−1^ m^−1^), T is the absolute temperature, and L is the Lorenz constant (2.44 × 10^−8^ W Ω K^−2^). The calculation was performed at room temperature (T = 298 K). Statistical analysis of the electrical conductivity values is reported as the mean of independent measurements with corresponding standard deviations. One-way ANOVA followed by Tukey’s test was performed to compare mean values, considering differences significant where *p* < 0.05. Figure 1 is a graphical representation of what was conducted and examined in this research paper.

## 3. Results and Discussion

### 3.1. Microstructural Characterization of the MA Powders

#### 3.1.1. Microstructural Analysis of the MA Powders

**Figure 2:**
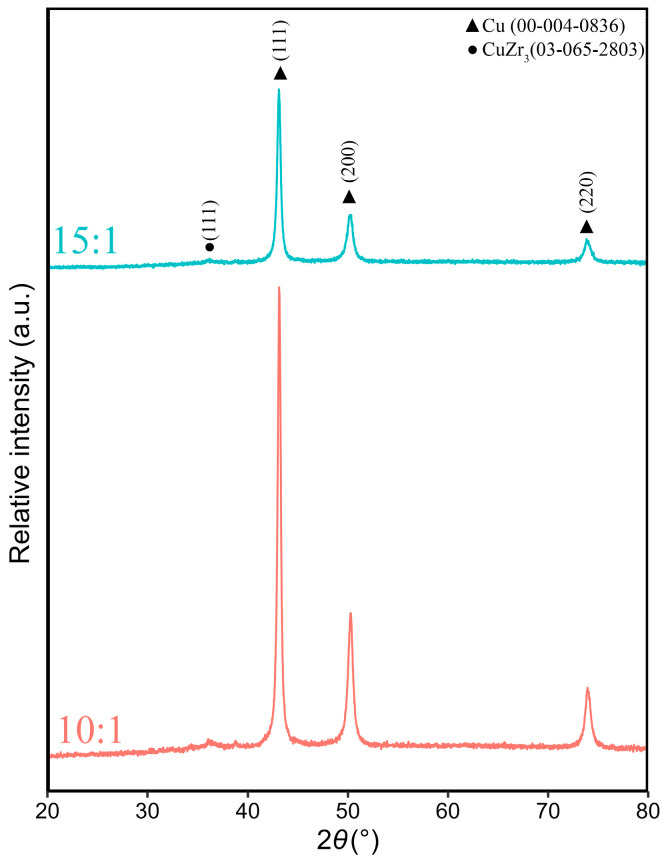
XRD patterns of the Cu–2Zr–0.6B (wt.%) powder mixture after 20 h of MA. In order, the first, with BPR 15:1, and the second, with BPR 10:1.

The XRD patterns of the powders are shown in Figure 2. As can be seen from the spectra, the starting powders do not contain any detectable oxides and do not belong to amorphous materials. The patterns show clear, high-intensity peaks of Cu (00-004-0836), while the peaks exhibiting lower intensity belong to CuZr_3_ (03-065-2803). A small amount of B was not detected due to instrumental limits. Furthermore, mechanical alloying did not result in the formation of ZrB_2_.

Mechanical alloying is a crucial step in the production process of these materials. It is also quite complex, and there are multiple variables that influence the outcome of the process. We have looked into: type of mill, milling time, milling speed (turning velocity-RPM), ball-to-powder weight ratio (BPR), type and size of milling media (balls), milling atmosphere, and process control agents (PCAs). For the purpose of process optimization, two fully controllable parameters were selected for detailed investigation: milling time and ball-to-powder weight ratio. Based on previous research [26], optimal results for Cu–Zr–B systems are typically achieved after 20 to 25 h of mechanical alloying. Therefore, a milling time of 20 h was chosen for this study. The second variable, BPR, was evaluated at two different ratios: 10:1 and 15:1, to assess their effect on the mechanical and structural parameters of the composite materials at hand.

**Figure 3:**
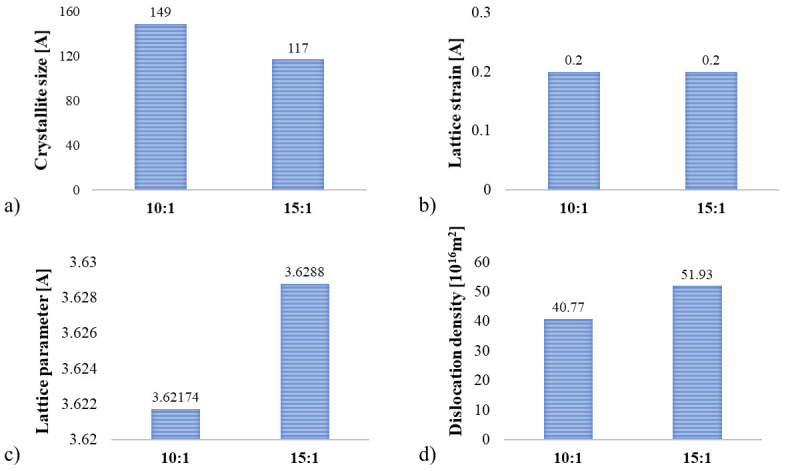
Structural parameters of MA Cu–2Zr–0.6B (wt.%) powders after 20 h of MA: (**a**) crystallite size, D (A), (**b**) lattice strain, ε (%), (**c**) lattice parameter *a* (A), and (**d**) dislocation density, ρ (m^−2^).

Structural parameters (crystallite size, lattice strain, and lattice parameter) of the mechanically alloyed powders were calculated from XRD data using Williamson–Hall analysis [27]. From Figure 3a, we can see that the crystallite size is higher for the BPR 10:1 sample compared to the BPR 15:1. Even though lattice strain values are same for both samples, as shown in Figure 3d, the decrease in crystallite size observed for the sample with a BPR 15:1 can be attributed to the more intense collisions between milling balls and powder particles where BPR is higher. Severe plastic deformation, as a consequence of high impact energy during these collisions, leads to a higher rate of defect creation, primarily dislocations, resulting in higher dislocation density. The increase in the FWHM value from 1.10 for BPR 10:1 to 1.80 for BPR 15:1 indicates a more pronounced broadening of the diffraction peaks, which is associated with a higher degree of plastic deformation and a greater number of defects in the crystal lattice. This is consistent with the calculated higher dislocation density for BPR 15:1, indicating that a higher ball-to-powder ratio leads to more intensive mechanical alloying and increased accumulation of defects within the powder structure.

Simultaneously, the lattice parameter shows a slightly higher value for the 15:1 BPR sample (Figure 3c), which is consistent with the assumption that intense plastic deformation and defect accumulation at higher BPR can lead to increased lattice distortion. In contrast, the lower lattice parameter for the 10:1 BPR sample may indicate a higher degree of recrystallized regions or the precipitation of supersaturated Zr and B atoms from the Cu matrix, both of which contribute to lattice contraction.

#### 3.1.2. Morphology and Size Analysis of the MA Powders

**Figure 4:**
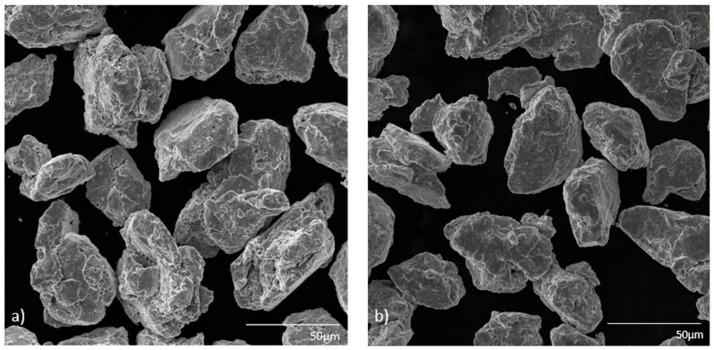
Microstructural SEM analysis of the mechanically alloyed Cu–2Zr–0.6B (wt.%) powders after 20 h of MA for (**a**) BPR 10:1 and (**b**) BPR 15:1.

The structure exhibits a highly refined and homogenous morphology, as can be seen in Figure 4, which is to be expected for prolonged mechanical alloying. All MA particles for both BPR 10:1 and BPR 15:1 observed are heavily deformed, fragmented, and cold-welded, resulting in irregular shapes and reduced particle size. Most particles are around 50–70 μm. This indicates that the repeated fracturing and welding processes, which are typical for mechanical alloying, have effectively reduced crystallite size, which was also confirmed by the XRD analysis. MIPAR and BET analyses were used to investigate in detail the differences between BPR 10:1 and BPR 15:1 MA powders. SEM images of MA powders provide evidence of significant surface roughness and agglomeration, which is expected due to the extended milling times and high-energy input. The formation of such structures suggests a high density of lattice defects, including dislocations and grain boundaries, consistent with the increased dislocation density observed as well in the XRD analysis.

**Figure 5:**
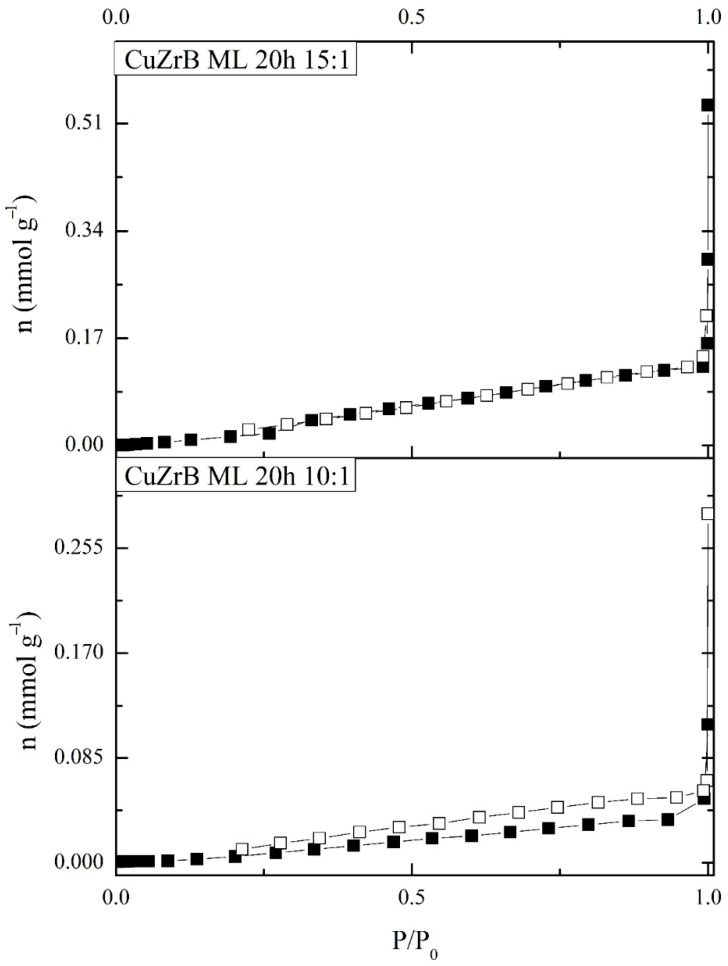
Nitrogen adsorption isotherm plot for Cu–2Zr–0.6B (wt.%) BPR 10:1 and Cu-2Zr-0.6B (wt.%) BPR 15:1 samples after 20 h of MA. Solid symbols—adsorption, open symbols—desorption.

Figure 5 depicts the nitrogen adsorption isotherms for the Cu-2Zr-0.6B (wt.%) BPR 10:1 and Cu–2Zr–0.6B (wt.%) BPR 15:1 samples. The specific surface areas (SBET), determined using the BET equation, were 2.42 m^2^/g and 3.74 m^2^/g, respectively. The isotherm profiles suggest that the Cu–2Zr–0.6B (wt.%) BPR 10:1 sample exhibits slightly higher porosity compared to Cu–2Zr–0.6B (wt.%) BPR 15:1. This trend, characterized by an increase in SBET values alongside a decrease in porosity, is consistent with the SEM micrographs. The observed differences can be attributed to the morphological characteristics of the samples: Cu–2Zr–0.6B (wt.%) BPR 10:1 consists of larger agglomerated particles, forming macropores between them, whereas Cu–2Zr–0.6B (wt.%) BPR 15:1 comprises more compact and finer particles, resulting in a higher surface area but reduced overall porosity.

**Figure 6:**
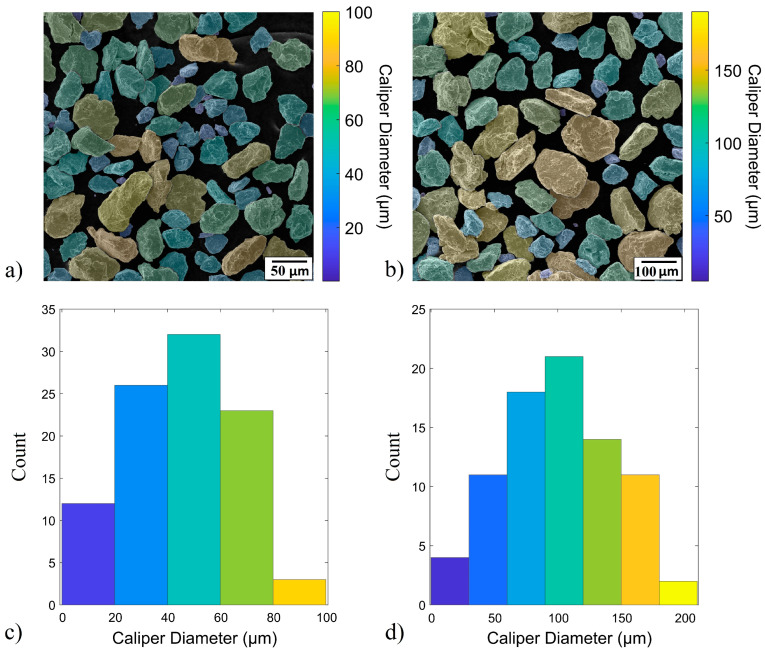

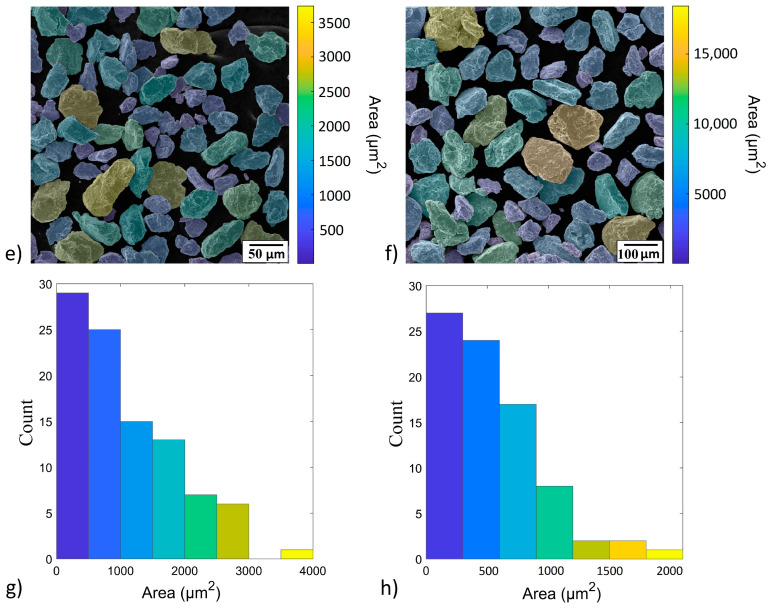
MIPAR results based on SEM images and particle size distribution histograms given in relation to caliper diameter (**a**–**d**), and area (**e**–**h**) for Cu–2Zr–0.6B powders after 20 h of MA. The (**a**,**c**,**e**,**g**) samples are with the BPR 15:1, and the (**b**,**d**,**f**,**h**) samples are with the BPR 10:1.

MIPAR software (version 4.5) was further employed to obtain quantitative data that is essential for understanding what happens during the MA process (Figure 6). Data about caliper diameter (the largest line length that fits across each feature), and area (area of each particle) have been provided for both the Cu–2Zr–0.6B (wt.%) BPR 10:1 and Cu–2Zr–0.6B (wt.%) BPR 15:1 samples. The caliper diameter results show that the BPR 10:1 sample (Figure 6b) shows a wider range of particle sizes, with some reaching up to 90 μm. In contrast, the BPR 15:1 sample (Figure 6a) mostly contains smaller particles, generally below 50 μm, which confirms that a higher BPR causes more intense particle size reduction. The BPR 10:1 powders include larger particles, some exceeding 60 μm, while the BPR 15:1 sample is made up of smaller, more consistent particles, most of which are under 30 μm. Looking at the area distribution, it is clear that the BPR 10:1 sample (Figure 6f) contains larger particles overall. Several particles exceed 3000 μm^2^, while the BPR 15:1 sample (Figure 6e) shows a much finer structure, with most particles having areas under 800 μm^2^. This suggests that higher BPR improves particle size reduction, producing a smaller and more uniform structure. The consistent contrast and absence of large, distinguishable particles suggest that the alloying between Cu, Zr, and B has been successfully achieved. This microstructure is favorable for the in situ formation of reinforcing phases like ZrB_2_ after sintering, which was the main goal of this study.

The histograms in Figure 6 provide a quantitative comparison of particle size metrics between samples processed with BPR 15:1 and BPR 10:1. All metrics support the trend that higher BPR (15:1) leads to smaller and more uniform particle sizes, while the lower BPR (10:1) results in a broader and coarser particle distribution. For caliper diameter, most particles in the BPR 15:1 sample (Figure 6c) fall between 20 and 60 μm, with a sharp decline beyond 80 μm, whereas the BPR 10:1 sample (Figure 6d) shows a broader distribution, peaking between 80 and 120 μm and going up to 180 μm, indicating a coarser structure. The area distribution highlights a similar contrast; the BPR 15:1 sample (Figure 6g) contains mostly small particles under 1500 μm^2^, while the BPR 10:1 sample (Figure 6h) includes a significant fraction of particles with areas exceeding 8000 μm^2^, going up to 18,000 μm^2^. Together, these results confirm that a higher BPR leads to more effective particle refinement and a more uniform microstructure.

### 3.2. Characterization of the Cu–ZrB_2_ Composites

Sintering parameters of the MA powders Cu–2Zr–0.6B (wt.%) BPR 10:1 and Cu–2Zr–0.6B (wt.%) BPR 15:1 samples are provided in Table 2. It is important to note that the theoretical density calculated based on the rule of mixtures for the composite used is 8.86 g/cm^3^. Based on the presented results, it is evident that the SPS and HP methods provide significantly higher densities compared to conventional cold pressing followed by sintering. The Cu–ZrB_2_ samples sintered by SPS reached densities of 8.15 g/cm^3^ (92%) and 8.0 g/cm^3^ (90%) for the 10:1 and 15:1 BPR samples, respectively, whereas the corresponding densities after conventional sintering were only 6.43 g/cm^3^ (73%) and 6.29 g/cm^3^ (71%). Samples consolidated by hot pressing also demonstrated excellent densification, achieving densities of 8.30 g/cm^3^ (94%) and 8.27 g/cm^3^ (93%) for the 10:1 and 15:1 BPR, respectively, which are slightly higher than those obtained by SPS and much higher than those from conventional sintering. The difference in density across these methods can be attributed to the fundamental differences in their mechanisms and process conditions [28,29,30,31,32]. As all compacts exhibit similar open porosity values, the CPS samples contain the highest total porosity. In agreement with the literature [13], the density was found to increase with sintering temperature, while open porosity decreased. Moreover, it should be noted that the SPS compacts obtained at 750 °C exhibited increased density compared to the literature data for SPS compacts sintered at 850 °C [13,14].

The SPS process uses a combination of high pressure and a pulsed electric current, which passes directly through the powder [29]. This creates rapid, localized heating at the contact points between particles (known as Joule heating), along with the formation of sparks or plasma discharges. The short sintering time (5 min) combined with a high applied pressure (35 Mpa) limits grain growth and reduces the formation of defects, which attributes to higher density and lower porosity as results have shown.

In the case of hot pressing, densification is achieved through the simultaneous application of elevated temperature and pressure over an extended period (2 h at 950 °C and 40 Mpa). This combination promotes enhanced atomic diffusion and particle rearrangement, improving interparticle bonding and reducing residual porosity compared to conventional sintering. Although the process lacks the direct electrical activation present in SPS, the continuous pressure and prolonged thermal exposure facilitate efficient consolidation and near-theoretical density. As a result, hot-pressed samples exhibit slightly higher densities than those produced by SPS, while still benefiting from refined microstructure and reduced defect formation.

On the other hand, conventional cold pressing and sintering rely solely on thermal diffusion at elevated temperatures over longer times (2 h) with no direct electrical input. Despite applying a much higher pressure during cold pressing (350 Mpa), the lack of electrical activation and slower diffusion kinetics during sintering limit particle packing and densification. This conventional process relies entirely on slower thermal diffusion mechanisms. As a result, the overall density is lower, and the levels of porosity are higher.

#### 3.2.1. Microstructural Analysis of the Cu–ZrB_2_ Composites

**Figure 7:**
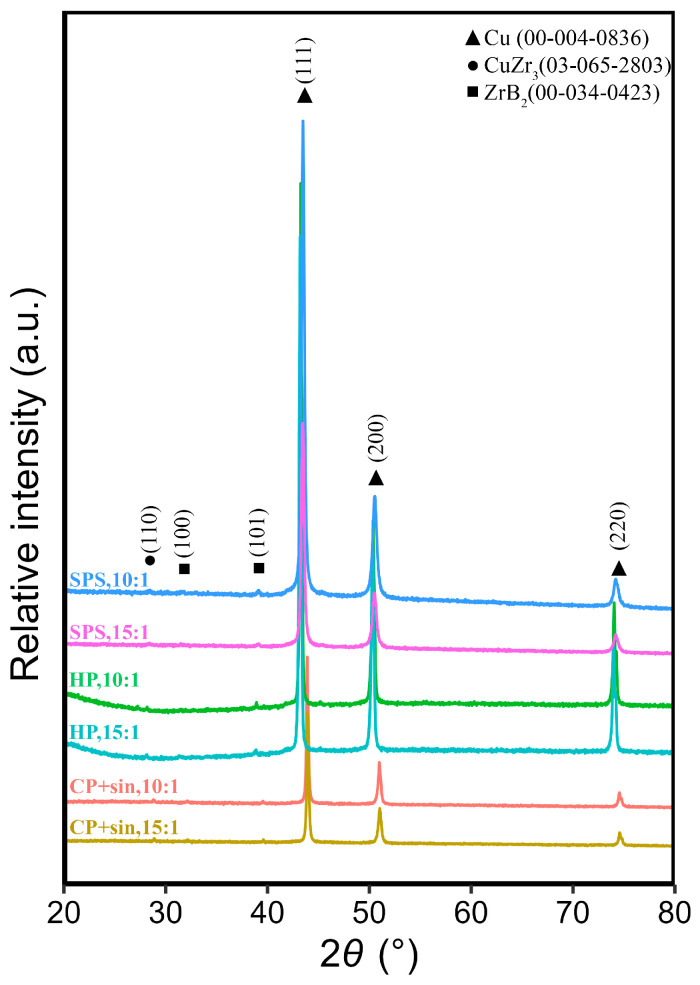
XRD patterns of Cu–ZrB_2_ samples, in order from the bottom to top: cold pressed and sintered sample with BPR 15:1 after 20 h of MA; cold pressed and sintered sample with BPR 10:1 after 20 h of MA; hot pressed sample with BPR 15:1 after 20 h of MA; hot pressed sample with BPR 10:1 after 20 h of MA; SPS sample with BPR 15:1 after 20 h of MA; SPS sample with BPR 10:1 after 20 h of MA.

The XRD analysis of the Cu–ZrB_2_ compacts (Figure 7) reveals the formation of ZrB_2_ particles around 3.5 vol.% calculated by the PDXL2 software (Rietveld refinement was conducted within the software, version 2.0.3.0). These PDXL results verified that the reaction was completed, as calculated values of formed ZrB_2_ were similar to the expected ones. An increase in the intensity and narrowing of Cu peaks was observed, indicating crystallite growth. CuKβ peak can be observed at 39° 2θ, corresponding to the (111) reflection, both in the MA and compact samples.

**Figure 8:**
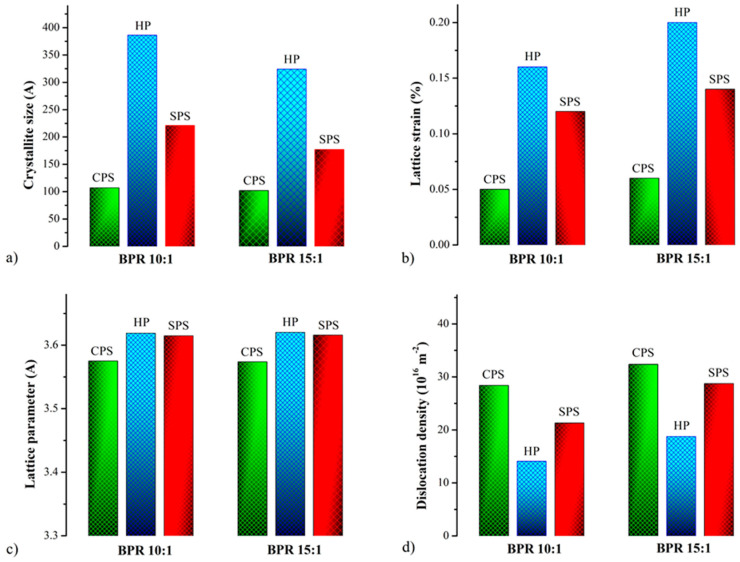
Structural parameters of Cu–ZrB_2_ samples after CPS, HP, and SPS: (**a**) crystallite size, *D* (A), (**b**) lattice strain, ε (%), (**c**) lattice parameter *a* (A), and (**d**) dislocation density, ρ (m^−2^).

Structural parameters for all three post-milling treatments have been provided in Figure 8. In all cases, increasing the ball-to-powder ratio results in a decrease in crystallite size and an increase in dislocation density, which is an expected outcome of higher milling energy. Moreover, the SPS-treated samples have significantly larger crystallite sizes (e.g., 221 A/at BPR 10:1) compared to the cold-sintered ones (e.g., 107 A at BPR 10:1), which suggests that the SPS process better preserves crystallinity. Furthermore, when the BPR is increased from 10:1 to 15:1 in SPS compacts, the crystallite size decreases from 221 A to 177 A, as a consequence of the effective consolidation of dominant smaller fragments obtained due to the higher input of mechanical energy. As a result, the dislocation density increases from 21.29 × 10^16^ m^−2^ to 28.72 × 10^16^ m^−2^, as there is a greater number of defects introduced by the more intense milling. The lattice parameter shows a slight increase, from 3.61471 A to 3.61575 A, which is consistent with lattice distortion caused by accumulated strain and dislocations owing to in situ formation and better distribution of reinforcing ZrB_2_ particles. The lattice strain value also increases from 0.12% to 0.14%, which further supports the conclusion that higher milling energy introduces more internal deformation prior to sintering. Similarly, dislocation densities have slightly lower values in the SPS samples (21.29–28.72 × 10^16^ m^−2^) than in the cold-sintered ones (28.39–32.62 × 10^16^ m^−2^), indicating that SPS may promote more dislocation or defect recovery. The lattice parameter shows a slight increase with milling intensity in SPS but a decrease in cold-sintered samples, implying differing responses to residual strain or thermal treatment. Meanwhile, lattice strain values are higher for both BPRs, 10:1 and 15:1, respectively, in SPS (0.12% and 0.14%) than in CPS (0.05% and 0.06%). It is important to conclude that the SPS process appears to better preserve crystallite size and reduce defect density compared to conventional sintering, likely due to its rapid densification and localized heating effects. When examining the hot-pressed samples, it was determined that values fall between SPS and CPS. The crystallite sizes are slightly reduced compared to SPS but remain notably larger than those produced by cold pressing and sintering. Likewise, the dislocation densities in HP compacts fall between those of SPS and CPS, reflecting improved defect recovery due to prolonged exposure to elevated temperature and pressure. The lattice parameter shows a slight increase with milling intensity in SPS but a decrease in CPS samples, implying differing responses to residual strain and thermal treatment. Hot pressing again shows intermediate values, likely due to enhanced diffusion kinetics over a longer time frame. Meanwhile, lattice strain exhibits slightly higher values for HP (0.16% and 0.2%) compared to SPS (0.12% and 0.14%) and CPS (0.05 and 0.06) for both BPRs, 10:1 and 15:1, respectively.

**Figure 9:**
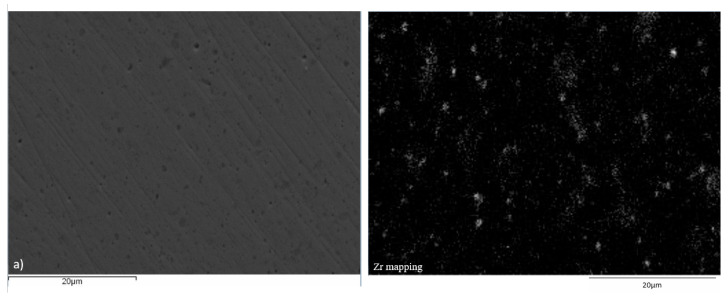

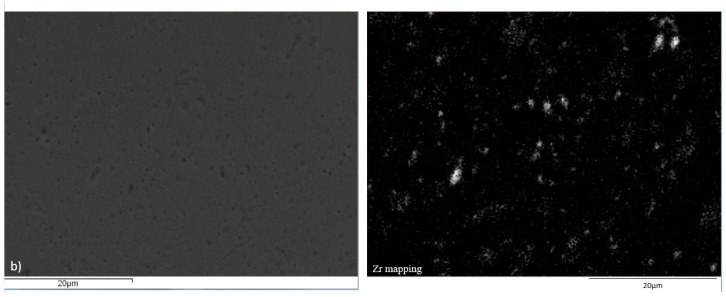
SEM-EDS analysis of the cold-pressed and sintered Cu–ZrB_2_ samples after 20 h of MA for (**a**) 10:1 BPR and (**b**) 15:1 BPR.

**Figure 10:**
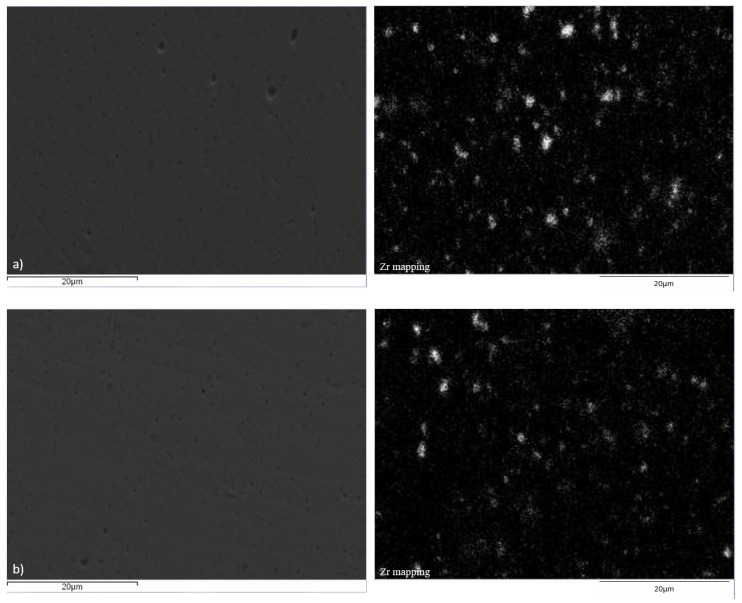
SEM-EDS analysis of the hot-pressed Cu–ZrB_2_ samples after 20 h of MA for (**a**) 10:1 BPR and (**b**) 15:1 BPR.

**Figure 11:**
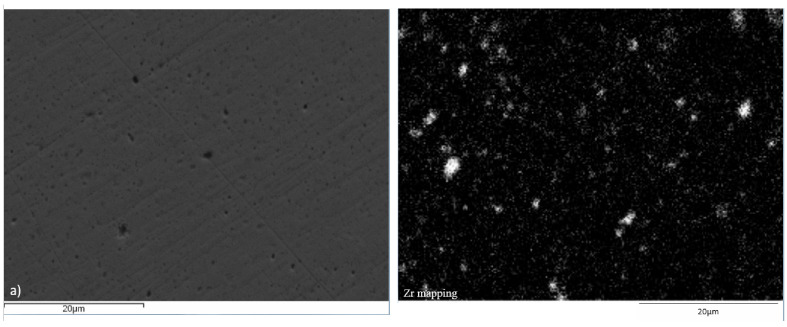

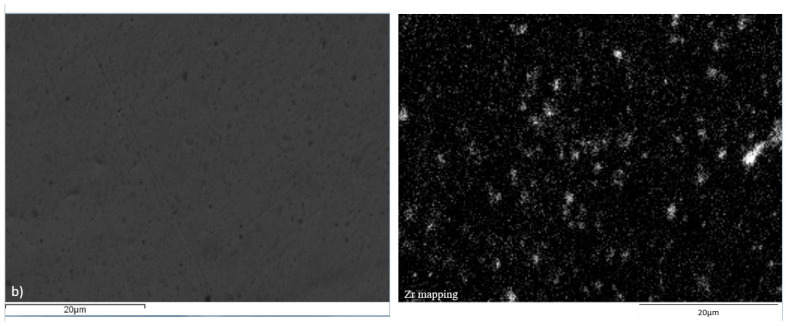
SEM-EDS analysis of the spark plasma sintered Cu–ZrB_2_ samples after 20 h of MA for (**a**) 10:1 BPR and (**b**) 15:1 BPR.

Microstructural and elemental analysis of the Cu–ZrB_2_ compacts was done by SEM-EDS (Figure 9, Figure 10 and Figure 11). Based on XRD results, it can be assumed that most of the Zr was involved in the formation of the ZrB_2_ particles during the consolidation process, so the distribution of the Zr particles presents the distribution of the ZrB_2_ particles inside the Cu matrix. As can be seen, the best distribution of the Zr particles was achieved after the SPS process (Figure 11), indicating the best distribution of the ZrB_2_ reinforcing particles in the copper matrix compared to HP (Figure 10) and CPS (Figure 9). The lowest distribution of Zr particles was achieved after the CPS, where non-uniform distribution within the grains can be observed due to the high porosity of the obtained compacts (Figure 9). SEM-EDS analysis of the high-density compacts (HP and SPS) displays better distribution of the Zr in the Cu matrix, which was reached in samples obtained from 15:1 BPR MA powders in all three cases, while the presence of agglomerated Zr particles was more dominant in compacts made of 10:1 BPR MA powders.

#### 3.2.2. Mechanical Properties of the Cu–ZrB_2_ Composites

The mechanical properties (elastic modulus, macro and nano hardness) of Cu–ZrB_2_ composites processed under different ball-to-powder ratios (BPR) and consolidation techniques are given in Table 3. The lowest hardness values were recorded for the samples produced by cold pressing followed by sintering, measuring 78.75 HV1 for BPR 10:1 and 81.97 HV1 for BPR 15:1. A significant increase in hardness was observed for the hot-pressed samples, which reached 152.63 HV1 (10:1) and 158.47 HV1 (15:1). The highest hardness values were achieved in the samples processed by spark plasma sintering (SPS), with 169.15 HV1 for BPR 10:1 and 176.70 HV1 for BPR 15:1. The cold-pressed and sintered samples exhibit the lowest hardness due to limited diffusion bonding and the presence of highest porosity. In contrast, hot pressing enhances hardness through improved particle rearrangement and partial plastic deformation, leading to higher density values and lower porosity. The highest hardness values achieved after SPS can be attributed to fine-grained microstructures and ZrB_2_ particle dispersion that occurs during rapid heating and short sintering cycles. These conditions promote grain boundary strengthening and uniform reinforcement distribution, both of which significantly contribute to the improved hardness. The obtained SPS compacts exhibit significantly higher hardness than those reported in the literature [13,14,15]. These findings indicate that through the optimization of mechanical alloying parameters, enhanced hardness can be achieved even at lower sintering temperatures using SPS.

When it comes to nanohardness and reduced elastic modulus values, SPS samples exhibited the highest nanohardness of 2.93 ± 0.51 GPa for BPR 15:1, 2.69 ± 0.12 GPa for BPR 15:1, followed by HP and CPS samples, which correlates with the macrohardness results, as expected. HP samples, with nanohardness values of 2.57 ± 0.03 GPa, BPR 10:1 and 2.38 ± 0.38 GPa, BPR 15:1, showed slightly lower values of the nanohardness compared to SPS values. Better distribution generates higher resistance to plastic deformation. Consequently, submicron ZrB_2_ particles, acting as obstacles to dislocation movements, increased dislocation density and, in turn, led to increased nanohardness. The reduced elastic modulus was highest in HP 10:1 (115.6 ± 2.99 GPa), reflecting strong grain cohesion. CPS samples showed comparable nanohardness values due to high dislocation density. On the other hand, their lower macrohardness values are influenced by the higher porosity of the bulk material, resulting in reduced overall mechanical performance.

#### 3.2.3. Electrical Conductivity of the Cu–ZrB_2_ Composites

**Figure 12:**
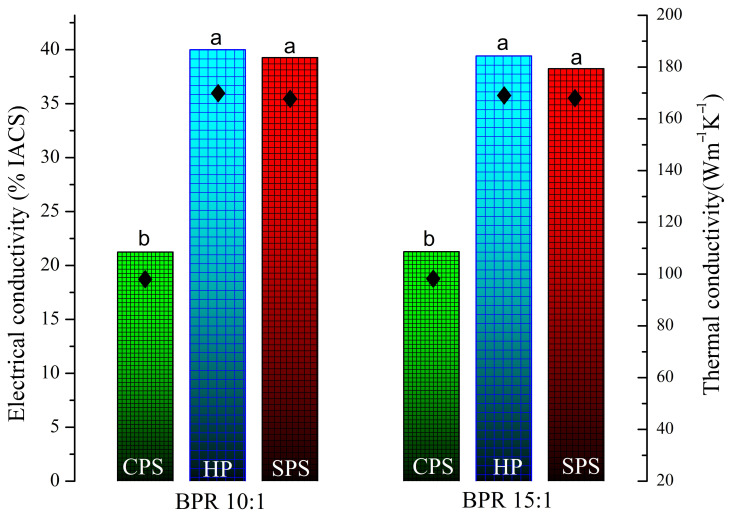
Electrical and thermal conductivity of Cu–ZrB_2_ BPR 10:1 and Cu–ZrB_2_ BPR 15:1 samples after cold pressing and sintering, hot pressing, and plasma sintering. One-way ANOVA followed by Tukey’s test was used to determine statistical significance (*p* < 0.05); different letters denote significant differences between groups.

The electrical conductivity (% IACS) of Cu–ZrB_2_ composites on the left axes, with corresponding thermal conductivity on the right axes, produced with two different ball-to-powder ratios (BPR 10:1 and 15:1) under various consolidation techniques, are displayed in Figure 12. The Cu–ZrB_2_ compacts obtained after cold pressing and sintering display the lowest conductivity values of 21.23 ± 0.12% IACS and 21.27 ± 0.03% IACS, respectively, which can be attributed to incomplete densification, residual porosity, and limited interparticle bonding. Moreover, the high dislocation density observed in CPS compacts contributes to their lower electrical conductivity. It is well known that dislocations act as scattering centers for electrons, leading to an increase in electrical resistivity. In contrast, both hot pressing (HP 10:1 39.99 ± 0.03% IACS, and HP 15:1 39.42 ± 0.06% IACS) and spark plasma sintering (SPS 10:1 39.26 ± 0.41% IACS, and SPS 15:1 38.24 ± 0.12% IACS) methods result in a significant improvement in electrical conductivity, reaching approximately 40% IACS for all conditions. This improvement is induced primarily due to enhanced densification, improved particle cohesion, and reduced porosity. Furthermore, these results indicate an enhancement in electrical conductivity compared to reported values [14], emphasizing the importance of optimizing mechanical alloying parameters before sintering. One-way ANOVA followed by Tukey’s test was performed to compare the mean values of electrical conductivity. Differences were considered statistically significant at *p* < 0.05. Results indicated significant differences between groups, with observed *p* values reaching *p* < .001. The compacts obtained after cold pressing and sintering exhibit relatively low thermal conductivity, with values of approximately 90 W m^−1^ K^−1^ for both BPR 10:1 and 15:1. A significant increase in thermal conductivity is observed after hot pressing, reaching 169 W m^−1^ K^−1^ for BPR 10:1 and 166 W m^−1^ K^−1^ for 15:1. Similarly, samples processed by SPS show high thermal conductivity values of 166 W m^−1^ K^−1^ (10:1) and 161 W m^−1^ K^−1^ (15:1). This improvement indicates that both hot pressing and SPS enhance thermal conductivity properties by promoting better densification and stronger interparticle bonding.

One of the major design challenges in developing high-performance Cu–matrix composites is to establish a material with excellent conductivity and improved mechanical properties. The use of reinforcing particles ZrB_2_ in the Cu matrix is a highly effective way to achieve an optimum combination of these electrical and mechanical properties, making Cu–ZrB_2_ composites suitable for demanding applications. In recent years, materials research in a field related to copper matrix composites has often engaged in overcoming the issue of lowering the conductivity due to the increasing hardness of Cu–matrix composites through innovative techniques to provide microstructure control and in situ formation of nano-reinforcements. The main goal of this study was to investigate the effect of mechanical alloying parameters and selected consolidation method on the electrical properties and mechanical behavior of the Cu–ZrB_2_ obtained composite materials. The obtained results revealed that SPS and HP are both suitable for manufacturing Cu–ZrB_2_ composites with excellent conductivity, where SPS provides slightly better mechanical properties. These findings strongly encourage further research focusing on the design of Cu–matrix composites with a lower content of micro- and nano-sized reinforcements produced by powder metallurgy.

## 4. Conclusions

The mechanically alloyed powders for 20 h Cu–2Zr-0.6B (wt.%) were consolidated via three processing routes (1. cold pressing and sintering, 2. hot pressing, and 3. spark plasma sintering). The results of all investigated samples are summarized below:

- XRD analysis of the MA powders revealed that 10:1 BPR showed higher values of crystallite size compared to 15:1 BPR, which is to be expected as the dislocation density for 15:1 BPR showed higher values as well. Further, MIPAR analysis of the size and shape of MA powders exposed that the BPR 10:1 possesses a wider range of particle sizes compared to the BPR 15:1 MA powders.
- Selection of the consolidation technique shows strong influence on the Cu–ZrB_2_ composite density where SPS (~91%) and HP (~93%) samples show significantly higher densities compared to CPS (~72%), mostly because SPS and HP apply pressure and heat simultaneously, enabling better densification and pore elimination, while CPS relies solely on heat, allowing more grain growth and pore retention.
- Microstructural analysis of the Cu–ZrB_2_ compacts displayed that across all three processing routes, increasing the ball-to-powder ratio consistently reduces crystallite size and increases dislocation density of compacts, with SPS preserving the largest crystallites and lowest defect densities due to rapid localized heating. CPS samples showed the most refined but defect-rich structures.
- The SPS samples possess the best mechanical properties with the highest values of the macro and nano hardness, followed by HP samples, while CPS samples exhibited significantly lower hardness values. CPS results in lower mechanical performance due to higher porosity and weaker particle bonding.
- HP and SPS samples show significantly higher electrical conductivity (~39% IACS) compared to CPS samples (~21% IACS), owing to their superior densification and lower porosity, which reduces phonon and electron scattering, thus enhancing transport properties.

The results demonstrate the potential of MA combined with SPS and HP to produce fine-grained Cu–ZrB_2_ composites with the highest density and improved electrical and mechanical properties for applications across aerospace, electrical–electronics, military, and automotive industries.

## Figures and Tables

**Table 1:** Sample abbreviations for all mechanically alloyed samples using all sintering methods.

Sample	Sintering Method	Abbreviation
1. Cu–ZrB_2_	SPS (Spark plasma sintering)	SPS, 10:1
2. Cu–ZrB_2_	SPS (Spark plasma sintering)	SPS, 15:1
3. Cu–ZrB_2_	Hot pressing	HP, 10:1
4. Cu–ZrB_2_	Hot pressing	HP, 15:1
5. Cu–ZrB_2_	Cold pressing and sintering	CPS, 10:1
6. Cu–ZrB_2_	Cold pressing and sintering	CPS, 15:1

**Table 2:** Sintering parameters of the Cu–ZrB_2_ BPR 10:1 and Cu–ZrB_2_ BPR 15:1 samples after spark plasma sintering (SPS), hot pressing, and conventional cold pressing, followed by sintering.

Composite Material	Temperature [°C]	Time [h]	Pressure [MPa]	Open Porosity [%]	Density [g/cm^3^]
SPS, 10:1	750	5 min	35	0.5907	8.15
SPS, 15:1	750	5 min	35	0.5932	8.0
HP, 10:1	950	2 h	40	0.5768	8.3
HP, 15:1	950	2 h	40	0.574	8.27
CPS, 10:1	1030	2 h	350	0.6243	6.43
CPS, 15:1	1030	2 h	350	0.6267	6.29

**Table 3:** Vickers macrohardness, nanohardness, and reduced elastic modulus values with the standard deviations of Cu–ZrB_2_ BPR 10:1 and BPR 15:1 samples after spark plasma sintering, hot pressing, and conventional cold pressing, followed by sintering.

Composite Material	Macrohardness, HV1	Nanohardness, H [GPa]	Reduced Elastic Modulus, Er [GPa]
SPS, 10:1	169.15 (±7.67)	2.69 (±0.12)	91.82779 (±8.66)
SPS, 15:1	176.70 (±1.95)	2.93 (±0.51)	105.7709 (±5.52)
HP, 10:1	152.63 (±3.66)	2.57 (±0.03)	115.60 (±2.99)
HP, 15:1	158.47 (±4.40)	2.38 (±0.38)	92.31 (±7.11)
CPS, 10:1	78.75 (±4.22)	2.86 (±0.84)	102.14 (±11.02)
CPS, 15:1	81.97 (±2.55)	2.24 (±0.08)	105.71 (±3.43)

## Data Availability

The original contributions presented in this study are included in the article. Further inquiries can be directed to the corresponding author.

## References

[1] Şap S., Uzun M., Usca Ü.A., Pimenov D.Y., Giasin K., Wojciechowski S. (2021). Investigation on microstructure, mechanical, and tribological performance of Cu base hybrid composite materials. J. Mater. Res. Technol..

[2] Wu J., Li Z., Gao Z., Wen G., Zhao Y., Li Y., Wu C., Nie H. (2024). Analysis of the mechanical, thermal and frictional behavior of Cu-sepiolite composite materials. Appl. Clay Sci..

[3] Li R., Guo E., Chen Z., Kang H., Wang W., Zou C., Li T., Wang T. (2019). Optimization of the balance between high strength and high electrical conductivity in CuCrZr alloys through two-step cryorolling and aging. J. Alloys Compd..

[4] Liang N., Liu J., Lin S., Wang Y., Wang J.T., Zhao Y., Zhu Y. (2018). A multiscale architectured CuCrZr alloy with high strength, electrical conductivity an thermal stability. J. Alloys Compd..

[5] Ye Y., Yang X., Wang J., Zhang X., Zhang Z., Sakai T. (2014). Enhanced strength and electrical conductivity of Cu–Zr–B alloy by double deformation–aging process. J. Alloys Compd..

[6] Butterworth G.J., Forty C.B.A. (1992). A survey of the properties of copper alloys for use as fusion reactor materials. J. Nucl. Mater..

[7] Wong-Ángel W.D., Téllez-Jurado L., Chávez-Alcalá J.F., Chavira-Martínez E., Verduzco-Cedeño V.F. (2014). Effect of copper on the mechanical properties of alloys formed by powder metallurgy. Mater. Des..

[8] Sharma S., Gajevic S., Sharma L., Mohan D., Sharma Y., Radojkovic M. (2025). Significance of the Powder Metallurgy Approach and Its Processing Parameters on the Mechanical Behavior of Magnesium-Based Materials. Nanomaterials.

[9] Suryanarayana C., Al-Aqeeli N. (2013). Mechanically alloyed nanocomposites. Prog. Mater. Sci..

[10] Suñol J.J. (2021). Mechanical alloying: Processing and materials. Metals.

[11] Taha M.A., Youness R.A., Zawrah M.F. (2019). Review on nanocomposites fabricated by mechanical alloying. Int. J. Miner. Metall. Mater..

[12] Shaik M.A., Golla B.R. (2020). Mechanical, tribological and electrical properties of ZrB_2_ reinforced Cu processed via milling and high-pressure hot pressing. Ceram. Int..

[13] Sulima I., Stepien M., Hyjek P., Boczkal S., Kowalik R. (2024). Mechanical, Corrosion and Wear Characteristics of Cu-Based Composites Reinforced with Zirconium Diboride Consolidated by SPS. Metals.

[14] Li C., Shi J., Li B., Ren W., Cao Z., Wei L., Wu D., Gao Y., Bai P., Chen Z. (2025). Interfacial characteristics, electrical conductivity, mechanical performance, and tribology behavior of ZrB_2_/Cu composites. Mater. Des..

[15] Sulima I., Stepien M., Hyjek P., Kowalik R. (2024). Effect of ZrB_2_ Content on the Properties of Copper Matrix Composite. Materials.

[16] Simić M., Ružić J., Božić D., Stoimenov N., Gyoshev S., Karastoyanov D., Stašić J. (2023). The influence of boron addition on properties of copper-zirconium alloys. Sci. Sinter..

[17] Simić M., Ružić J., Božić D., Stašić J. (2023). The effect of ball-to-powder ratio on the mechanical and structural properties of CuZrB composite materials fabricated by powder metallurgy. Metall. Mater. Data.

[18] Ding H., Miao W., Huang X., Liu Q., Fan X., Wang H., Chu K., Li C. (2020). Influence of Al addition on microstructures of Cu–B alloys and Cu–ZrB_2_ composites. Trans. Nonferrous Met. Soc. China.

[19] Li B., Gao Y., Li C., Cao Z., Yao X., Wu D., Bai P., Chen Z. (2025). Microstructural characteristics, electrical conductivity and mechanical properties of Cu matrix composites reinforced with dual-phase borides. J. Mater. Res. Technol..

[20] Zhang Z., Sheng Y., Xu X., Li W. (2015). Microstructural features and mechanical properties of in situ formed ZrB_2_/Cu composites. Adv. Eng. Mater..

[21] (2011). PDXL.

[22] Yun P., Fu H., Zhang H., Sun J., Zhao M., Xie J. (2025). Rapid design of high-end copper alloy processes combining orthogonal experiments, machine learning, and Pareto analysis. J. Mater. Res. Technol..

[23] Klabunde K.J. (2001). Nanoscale Materials in Chemistry.

[24] Roshan M.R., Mirzaei M., Jahromi S.A.J. (2013). Microstructural characteristics and tensile properties of nano-composite Al 2014/4 wt.% Al_2_O_3_ produced from machining chips. J. Alloys Compd..

[25] (2012). Standard Test Method for Resistivity of Electrical Conductor Materials.

[26] Simić M., Luković A., Jovanović G. (2024). Quantitative analysis of mechanically alloyed CuZrB powders. Metall. Mater. Data.

[27] Himabindu B., Latha Devi N.S.M.P., Rajini Kanth B. (2021). Microstructural parameters from X-ray peak profile analysis by Williamson-Hall models: A review. Mater. Today Proc..

[28] Onyishi H.O., Oluah C.K. (2020). An overview of the state of the art and applications of sintered metals. IOSR J. Mech. Civ. Eng..

[29] Mitra S., Maiti T. (2019). Thermoelectric materials synthesized by spark plasma sintering (SPS) for clean energy generation. Spark Plasma Sintering of Materials.

[30] Khoshghadam-Pireyousefan M., Mohammadzadeh A., Heidarzadeh A., Brabazon D. (2021). Fundamentals of spark plasma sintering for metallic, ceramic, and polymer matrix composites production. Encyclopedia of Materials: Composites.

[31] Sharma N., Alam S.N., Ray B.C. (2019). Fundamentals of spark plasma sintering (SPS): An ideal processing technique for fabrication of metal matrix nanocomposites. Spark Plasma Sintering of Materials.

[32] Rogers S., Dargusch M.S., Otte J., Kent D. (2024). Advancing sintering and matrix-reinforcement interaction in Al/AlN metal matrix composites through use of novel AlN reinforcement. Mater. Sci. Eng. A.

